# Short-Record-Based Extreme Wind Simulation

**DOI:** 10.6028/jres.099.037

**Published:** 1994

**Authors:** Edmond D. H. Cheng, Arthur N. L. Chiu

**Affiliations:** Department of Civil Engineering, University of Hawaii at Manoa, Honolulu, Hawaii 96822

**Keywords:** extreme wind speed, Gumbel distribution, Markov chain model, simulation, transition probability matrix

## Abstract

In order to utilize limited historical wind records for estimating extreme wind speeds for natural hazards damage mitigation, a Markov chain model for generating long-term annual extreme winds, on the basis of short-term records, is investigated. Basically, this simulation model consists of three components. They are State of wind speeds, wind speed distribution functions, and transition probability matrices.

The basic strategy of our simulation model is to generate the time series of hourly wind speeds in parts: for those winds associated with well-behaved climates and those with extreme winds. Applications of this model to generate long-term extreme winds, on the basis of short records at Houston Intercontinental Airport of Texas, arc demonstrated.

## 1. The Model

Recent efforts have been made to improve the methods of utilizing short-term wind records [1,2,3,4]. Our contribution to this endeavor is the development of stochastic simulation models which generate hourly wind data on a daily cycle basis [5,6].

The three essential elements in the Markov chain model are: state of wind speeds, wind speed distribution functions, and transition probability matrices.

### 1.1 State of Wind Speeds

In the simulation process for a given wind site, the first step is to divide the entire range of observed wind speeds into a finite number of states. This is performed with reference to the probability histogram derived from the observed wind data for that site. A computer program called WSTAT was developed for performing this task.

### 1.2 Distribution Functions

The second basic element involves the wind speed distribution functions, viz., the probability density functions (PDF) and the cumulative distribution functions (CDF) of wind speeds in various states. In this paper, four types of PDFs are utilized to fit a wind speed histogram, viz., uniform, linear, exponential and Weibull distribution functions. The exponential and Weibull distributions are exclusively reserved for the last state in which extreme winds are involved.

### 1.3 Transition Probability Matrices

The transition probability *P_ij_* is actually a conditional transition probability of wind speed *v_τ_* going from one state *i* at hour *τ* to wind speed *v*_τ+1_ of state *j* at hour *τ* + 1 or

$$P_{ij}=p\left(v_{\tau +1}=j|v_{\tau }=i\right).$$ (1)

With *m* states determined, an *m* × *m* transition probability matrix *PM* can be determined as

$$PM=\left[p_{ij}\right]\;\text{for}\;i,j=1,2,\ldots ,m$$ (2)

in which, *P_ij_* have the following properties:

$$\sum_{j=1}^{m}p_{ij}=1,\;\text{for}\;i=1,2,\ldots ,m$$ (3)

and

$$p_{ij}>0,\;\text{for all}\;i\;\text{and}\;j.$$ (4)

In this paper, a day is divided into several periods. Similarly, the variation of mean monthly wind speeds is accounted for by grouping consecutive months with similar wind speed trends into a number of seasons for a year. If the number of periods in a day and the number of seasons in a year are *R* and *S*, respectively, then there will be *R* × *S* transition probability matrices in the simulation process. A typical transition probability matrix for a given period *r* and season *s* can be expressed as

$$PM\left(s,r\right)=\left[{p_{ij}}^{s,r}\right]$$ (5)

where *s* = 1,2,…,*S*, and *r* = 1,2,…,*R*. A computer program called WTPM was developed for calculating the *PM*(*s,r*).

## 2. Simulation Procedures

Some sequential steps of generating hourly wind data points at a given site are briefly described as follows:

1. Divide the historical wind data into subsets so that program WSTAT may be executed.
2. Define *R*, number of periods in a day, and *S*, number of seasons in a year, so that program WTPM is activated.
3. Calculate PDFs and CDFs of the historical data.
4. By means of Eq. (5), compute *PM*(*s*,*r*), the transition probability matrix of period *r* in season *s*. A total of *R × S* transition probability matrices will be obtained.
5. Determine the state of the succeeding hour’s wind speed. For any given wind speed in state *i* of this current hour (with specified period *r* and season *s*), the succeeding hour’s wind speed state interval “*k*” can be determined.
6. Determine the value of the succeeding hour’s wind speed. With the state *k* determined in Step 5, the simulated wind speed for the succeeding hour can thus be obtained.

Repeat steps 5 and 6 until a desired period of simulation is attained. A computer program called WSIM was developed for generating the hourly wind data.

## 3. Markov Property and Stationary Tests

In order to substantiate the major assumptions made earlier, a test must be performed of the Markov property, i.e., the existence of dependency between two adjacent hourly wind speeds. This simulation technique is only applicable to stationary time series; the intended simulation model is a stationary first order Markov chain. Consequently, a test of stationarity of the historical wind speed times series is necessary prior to the acceptance of the simulated results. Anderson and Goodman’s method [7] was used in performing these tests in Sec. 4.

## 4. Application

The simulation model based on the described procedures is applied to wind data collected at the Houston Intercontinental Airport in Texas. In this illustration, three periods in a day (1:00 a.m.–9:00 a.m., 10:00 a.m.–7:00 p.m., and 8:00 p.m.–mid-night) and two seasons in a year (November–May and June–October) were considered. The periods of a day were decided from the averaged diurnal wind speeds at the site. Therefore, by using Eq. (5), six transition probability matrices were calculated.

Based on the 20 years (January 1973 to September 1992) of hourly wind records available at Houston Intercontinental Airport, eight simulation runs were made. Each run generated 100 years of hourly wind speeds. Historical record periods for the eight runs are: the first 5 years (January 1973–December 1977); the second 5 years (January 1978–December 1982); the third 5 years (January 1983–December 1987); the fourth 5 years (January 1988–September 1992); the first 10 years (January 1973–December 1982); the second 10 years (January 1983–September 1992); the first 15 years (January 1973–December 1987); and all 20 years (January 1973–September 1992).

The annual extreme wind speeds of the historical data (1963–1990), and of eight sets, each 100 years long, of simulated hourly data, are plotted on Type I probability paper (Fig. 1). Using the Gumbel line fitted to the historical data as the reference, the performance of the simulation model is summarized in Table 1. As shown in this table, the deviations of the simulated 25-year, 50-year, or 100-year wind speeds from the reference Gumbel line were measured by *S_v_*, Cramer-Rao’s standard deviation of the inherent sampling error of the historical records [8,9]. As indicated in Table 1, the differences between the simulated annual extreme wind speeds and the values obtained from the reference Gumbel line of historical data at 25 year or 50 year or 100 year recurrence intervals are all smaller than one *S_v_*. This result is very encouraging.

The plots of cumulative distribution functions for the historical as well as the 100 year generated records at Houston Intercontinental Airport are presented in Fig. 2. As shown in this figure, the curves derived from the generated records closely match those of the historical records, which implies that the characteristics of the historical wind speeds at Houston Intercontinental Airport were adequately represented.

## 5. Conclusions

A procedure for predicting extreme wind speeds at a location along the Gulf Coast is demonstrated. The results obtained from the application of this model are very encouraging. Although 20 years of data were available for the particular station in the illustration, computer simulation runs were made on the basis of 20 year, 15 year, 10 year, and 5 year database. It has been shown that it is not necessary to have 20 years of continuous data and that even a 5 year record is adequate for showing good comparison between the simulated results and historical data. Further research effort is being undertaken at the University of Hawaii at Manoa to study the applicability of this method to other stations along the Gulf Coast and other parts of the continental United States.

## Figures and Tables

**Fig. 1a, b:**
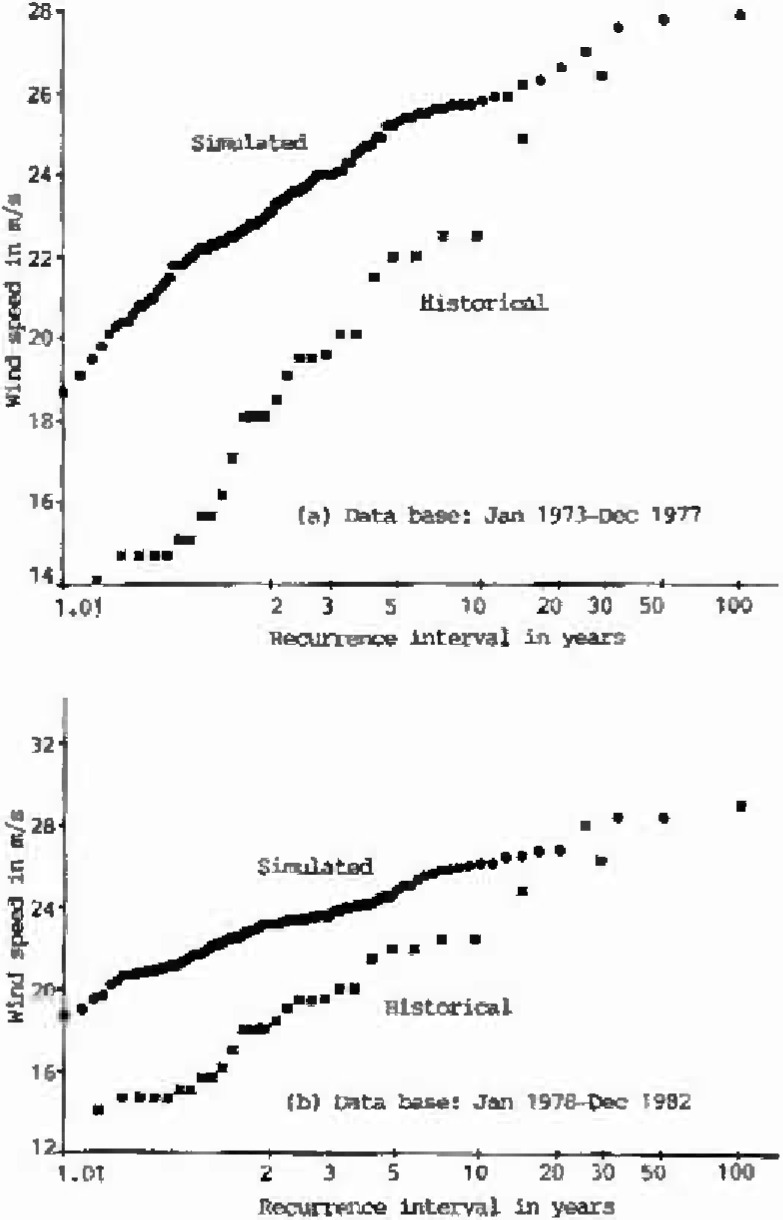
Type I distributions of historical and simulated annual extreme wind speeds at Houston Intercontinental Airport at 10 m above ground level.

**Fig. 1c, d:**
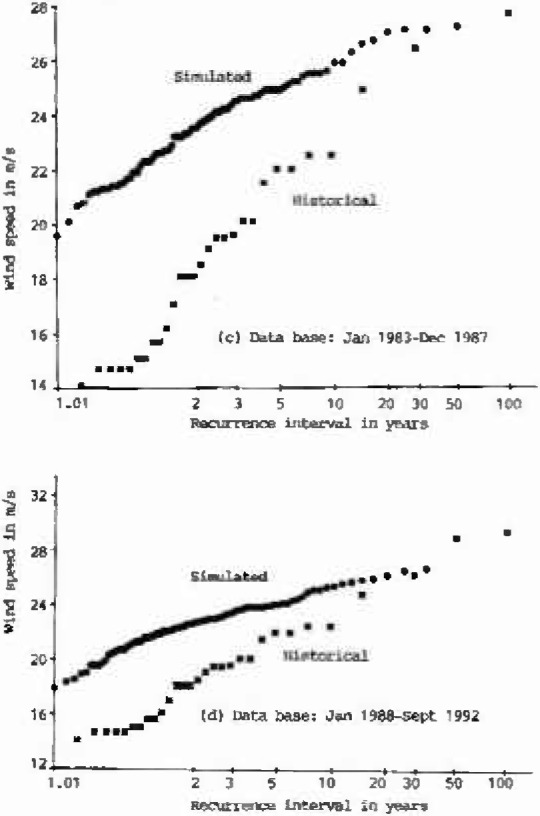
Type I distributions of historical and simulated annual extreme wind speeds at Houston Intercontinental Airport at 10 m above ground level.

**Fig. 1e, f:**
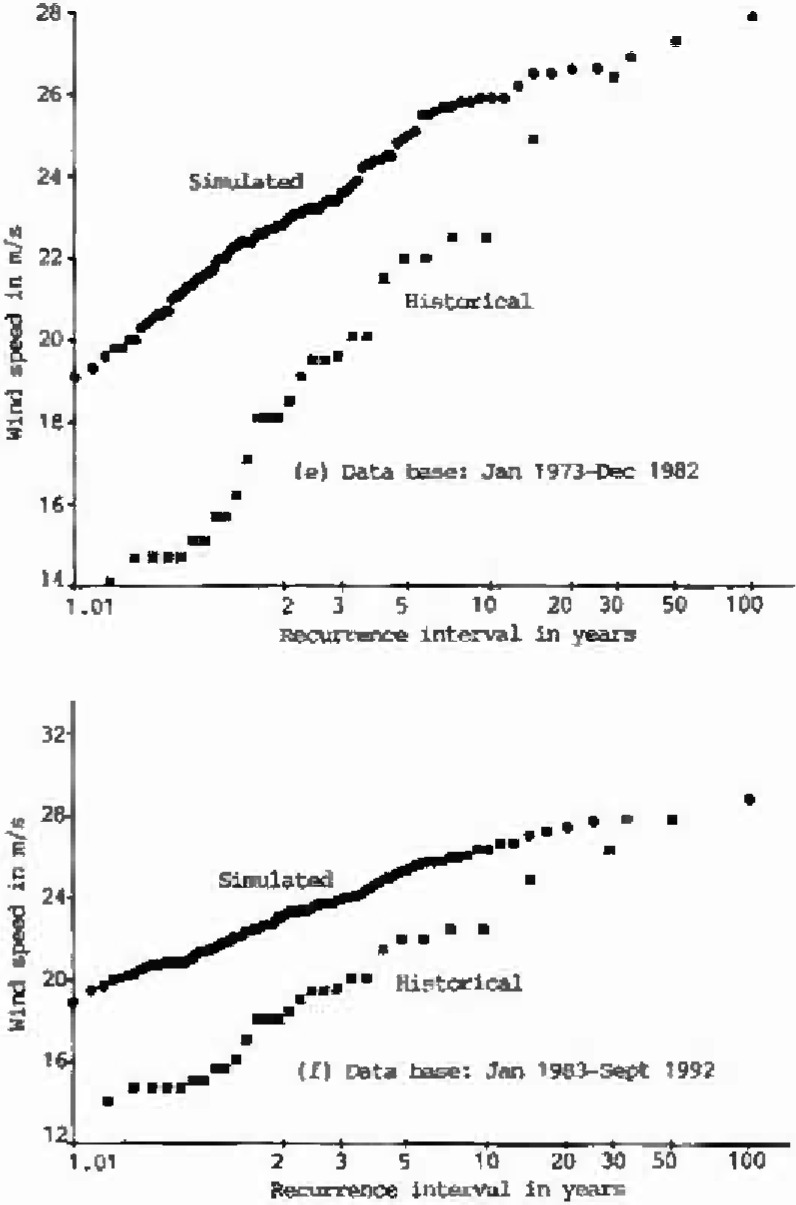
Type I distributions of historical and simulated annual extreme wind speeds at Houston Intercontinental Airport at 10 m above ground level.

**Fig. 1g, h:**
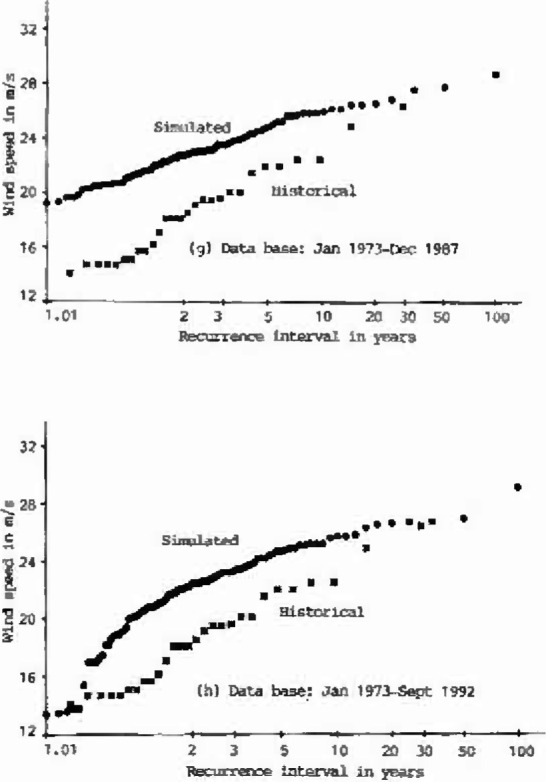
Type I distributions of historical and simulated annual extreme wind speeds at Houston Intercontinental Airport at 10 m above ground level.

**Fig. 2a, b:**
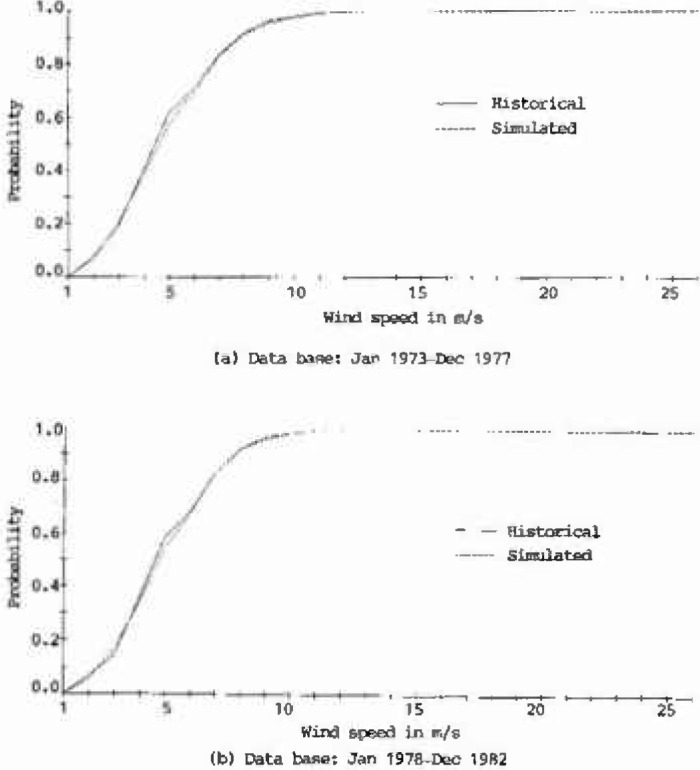
Cumulative distribution functions of historical and simulated hourly wind speeds at Houston Intercontinental Airport at 10 m above ground level.

**Fig. 2c, d:**
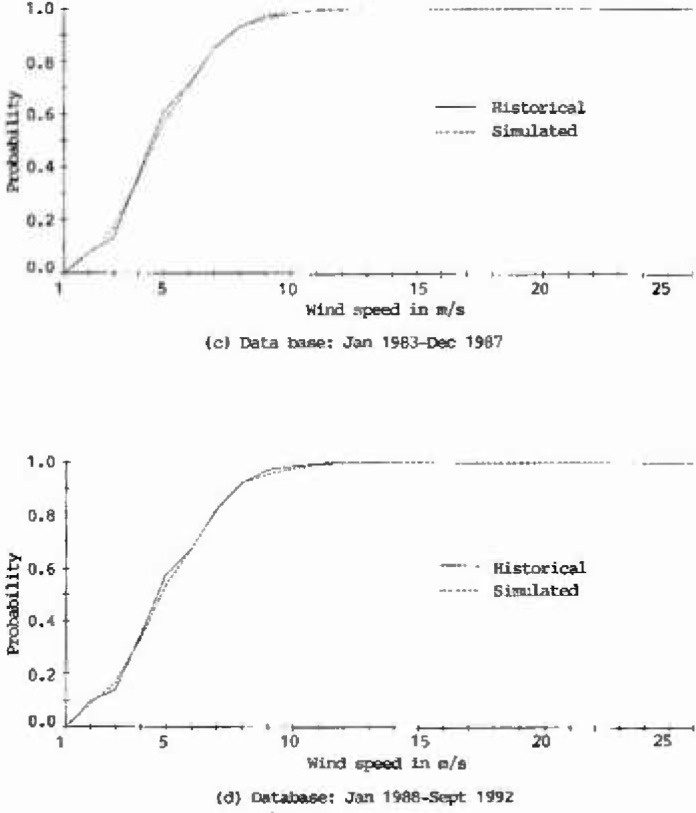
Cumulative distribution functions of historical and simulated hourly wind speeds at Houston Intercontinental Airport at 10 m above ground level.

**Fig. 2e, f:**
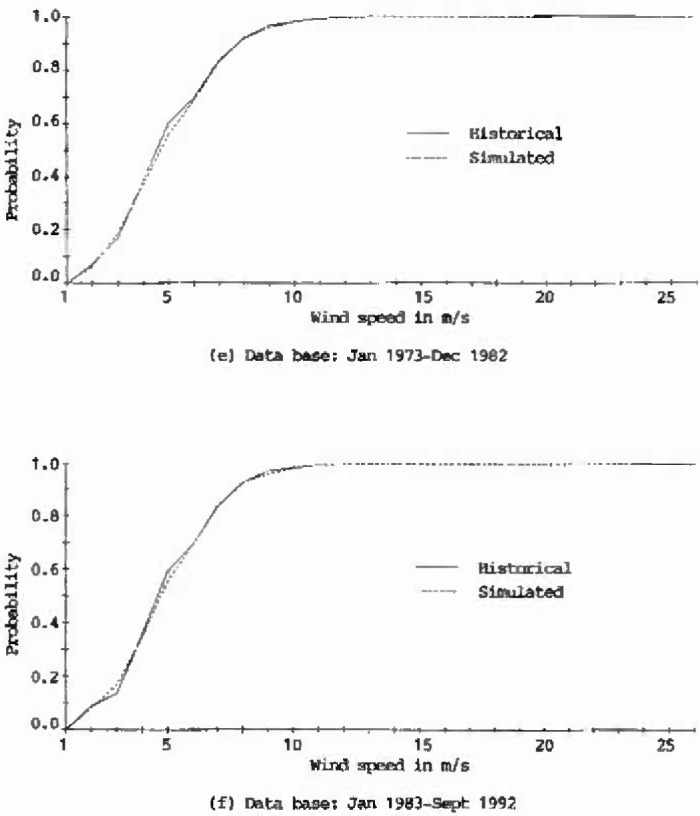
Cumulative distribution functions of historical and simulated hourly wind speeds at Houston Intercontinental Airport at 10 m above ground level.

**Fig. 2g, h:**
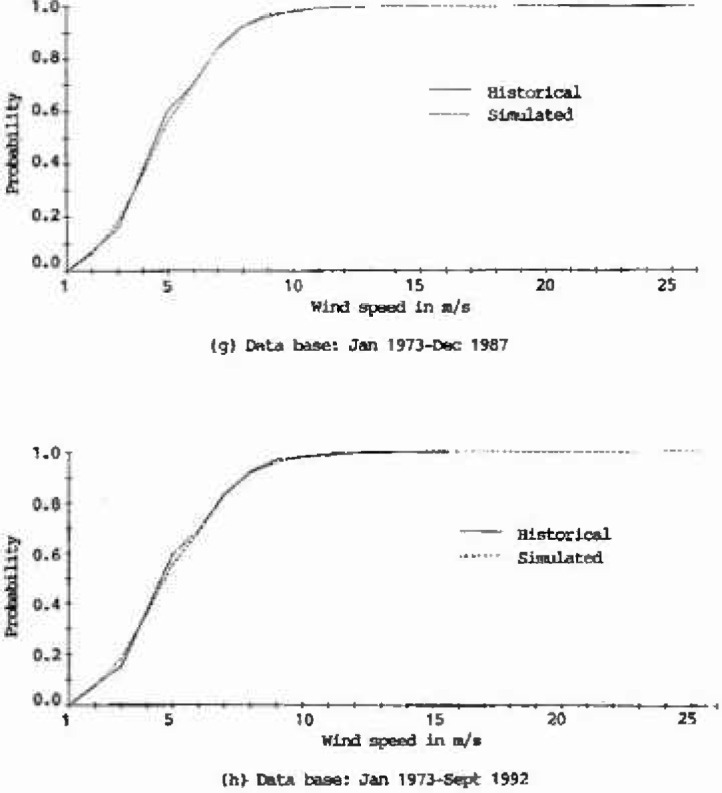
Cumulative distribution functions of historical and simulated hourly wind speeds at Houston Intercontinental Airport at 10 m above ground level.

**Table 1:** Estimated annual extreme wind speeds from historical records and from simulation methods at Houston Intercontinental Airport

Data period	Recurrence interval (in years)	*V*_a_	*S*_v_	*V*_s_	$\frac{V_{a}-V_{s}}{S_{v}}$
1963–1990	25	26.6	1.78		
	50	28.8	2.13		
	100	30.9	2.48		
1/1973–12/1977	25			27.6	−0.56
	50			28.7	0.05
	100			29.9	0.40
2/1978–12/1982	25			27.8	−0.67
	50			29.1	−0.14
	100			30.3	0.24
1/1983–12/1987	25			27.4	−0.45
	50			28.4	0.19
	100			29.4	0.60
1/1988–9/1992	25			27.4	−0.45
	50			28.6	0.09
	100			29.8	0.44
1/1973–12/1982	25			27.4	−0.45
	50			28.6	0.09
	100			29.7	0.48
1/1983–12/1987	25			28.1	−0.84
	50			29.4	−0.28
	100			30.7	0.08
1/1973–12/1987	25			27.6	−0.56
	50			28.8	0.00
	100			30.0	0.36
1/1973–9/1992	25			27.9	−0.73
	50			29.6	−0.38
	100			31.3	−0.16

*V*_a_ = extreme wind speed from annual series in m/s and 10 m above ground level.

*V*_s_ = simulated extreme wind speed in m/s and 10 m above ground level.

*S*_v_ = Cramer-Rao’s standard deviation of inherent sampling error of historical records.
